# Interplay of miR-243 and microbial interactions in aging and transgenerational immunity

**DOI:** 10.1080/19490976.2025.2537750

**Published:** 2025-07-28

**Authors:** Arun Kumar, Dilawar A. Mir, Mojibur R. Khan

**Affiliations:** a Molecular Biology and Microbial Biotechnology Laboratory, Division of Life Sciences, Institute of Advanced Study in Science and Technology (IASST), Guwahati, India; b Laboratory of Molecular Basis of Aging and Rejuvenation, Institute of Biochemistry and Biophysics, Polish Academy of Sciences (IBB-PAS), Warsaw, Poland; c School of Gerontology, University of Southern California, Los Angeles, CA, USA; d Academy of Science and Innovative Research (AcSIR), Ghaziabad, India

**Keywords:** miR-243, microRNA, proteostasis, gut microbiota, DAF-16, autophagy-lysosome pathway, transgenerational immunity

## Abstract

MicroRNAs (miRNAs) function in post-transcriptional regulation of gene expression and influence numerous biological processes. This commentary and view provide integrated insights from Kumar et al. (2024) and Gabaldon et al. (2020), emphasizing the role of miR-243 in promoting longevity and stress adaptation in response to microbial cues. However, further research is necessary to identify precise molecular targets of miR-243, comprehend its interactions with other miRNAs, validate its conservation in higher organisms, and to explore its potential toward targeted therapeutic strategies against aging and stress-related diseases.

## Introduction

Aging is a complex biological process shaped by various genetic, environmental, and microbial factors.^1^ Recent studies have highlighted the significant role of gut microbiota in influencing the host physiology and aging process across various organisms, including *Caenorhabditis elegans*, rodents and humans.^1^ Among the diverse host factors influenced by microbial signals, microRNAs (miRNAs) are small non-coding RNAs involved in regulation of post-transcriptional gene expression, which have recently garnered lots of attention.^2^ Notably, the Nobel Prize in Physiology or Medicine in 2025 was awarded for uncovering the role of microRNA in *C. elegans*, which provided the foundation for understanding miRNA-mediated gene expression.^3^ Although this Nobel-winning work was not directly related to microbial cues, but it motivated a wave of discoveries demonstrating how particular miRNAs integrate microbial and environmental signals to modulate aging, immunity, and stress responses. These developments have thus positioned miRNAs as a key mediator in host-microbe interaction and adaptive physiology.

Previous research showed several mechanisms through which microbiota can modulate host’s miRNA expression profile.^4^ For instance, microbial metabolites, including short-chain fatty acids (SCFAs) released by microbiota, can influence host’s epigenetic landscape through inhibition of histone deacetylases (HDACs), and alter the miRNA expression.^5^ Similarly, microbe-associated molecular patterns (MAMPs) can activate host’s pattern recognition receptors (e.g. Toll like receptors), that triggers downstream signaling pathways, which in turn remodels chromatin and alters transcriptional activation of immune-associated miRNAs.^6^ Notably, bacterial lipopolysaccharides (LPS) activate TLR4 that upregulates the expression of miR-146a and miR-155, regulating the host’s inflammatory responses.^6^ Moreover, bacterial small RNAs and bacterial outer membrane vesicles have also shown interference with the host’s miRNA processing machinery.^7,8^ Additionally, microbial cues have been found to play a role in the transmission of adaptive immune traits (transgenerational inheritance),^9^ although it has been poorly linked with miRNA.

Within this dynamic regulatory landscape, miR-243 has emerged as a promising key candidate at the intersection of microbiota sensing and modulating host’s aging and stress adaptation pathways.^10,11^ Our particular selection of miRNA-243 was guided by two independent methodologically rigorous, and unbiased important studies, viz. Kumar et al.^10^ and Gabaldon et al.^11^. Both the studies employed high-throughput miRNA sequencing and functional assays to identify miRNAs regulated by exposure to beneficial or pathogenic microbes in *C. elegans*. Among the miRNAs, miR-243 has emerged as the consistent hit and was strongly upregulated after following exposure to both bacterial probiotic (i.e. *Levilactobacillus brevis* MKAK9) and pathogens (i.e. *Salmonella enterica* serovar Typhimurium MST1 and *Pseudomonas aeruginosa* PAO1), indicating its potential biological role as a downstream effector of bacterial signal sensing pathways. Unlike other miRNAs with inconsistent, transient, or broad responses to microbial stimuli, miR-243 demonstrated a robust, specific, and functionally important expression pattern, playing a role in proteostasis, stress adaptation and transgenerational immunity.

Importantly, aging process is greatly influenced by changes in the profile of gut microbiota, and recent research linked microbial cues to both longevity and intergenerational plasticity.^12,13^ Age-associated changes in microbiota composition, characterized by increased pathobiont and decreased diversity, contribute to impaired barrier function, chronic inflammation, and metabolic dysregulation, resulting in accelerated aging process and phenotypes.^14^ This microbiota alteration can also affect the host’s miRNA expression profile, generate a feedback look for further influencing stress responses and aging trajectories.^15^ Intergeneration transfer of these microbiota-induced signals shows another important dimension, as parental microbial treatment can transmit the adaptive response benefits to progeny through epigenetic mechanisms.^16^ miR-243 may serve as the central regulatory axis to integrate microbial environment, aging process, and epigenetic memory that promote longevity and stress resilience.

This commentary integrates the finding from Kumar et al.^10^ and Gabaldon et al.^11^, which collectively offer complementary insights into the role of miR-243 in *C. elegans*. These studies were chosen based on their rigorous methodology, shared common molecular mediator (miR-243), and distinct yet aligning physiological outcome (probiotic versus pathogen exposure). To contextualize these studies, we have included literature on aging biology, epigenetic inheritance, and microbiota-microRNA interactions.

## Key findings from Kumar et al.^10^: miR-243 in proteostasis and the autophagy-lysosome pathway

**Figure 1.**
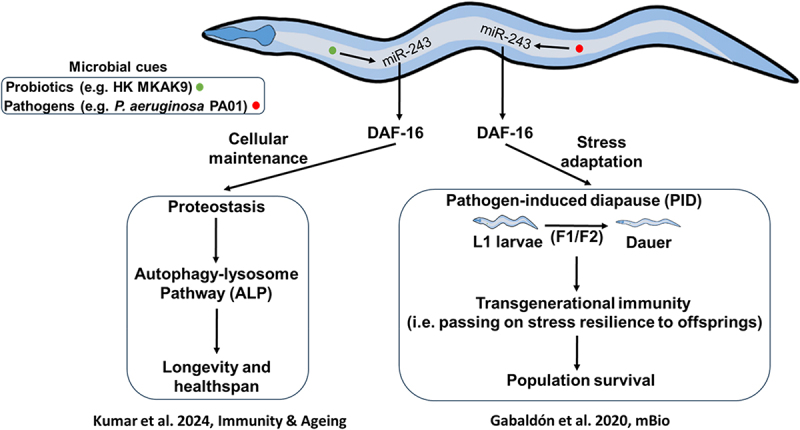
miR-243 regulates longevity, healthspan, and transgenerational immunity in *C. elegans*.

Kumar et al.^10^ provided interesting insights into the role of miR-243 in improving longevity of *Caenorhabditis elegans*.^10^ Their results showed how probiotic interventions, such as heat-killed *Levilactobacillus brevis* MKAK9 (HK MKAK9) and its exopolysaccharide (EPS) induced the expression of miR-243, which further modulated important cellular processes promoting healthy aging. Mechanistically, the upregulation of miR-243 was found to partially regulate the insulin-like signaling (ILS) pathway, especially in a DAF-16-dependent manner. DAF-16 is a transcription factor that generally translocates to the nucleus upon activation and upregulates the key genes required in stress resilience, metabolic homeostasis, and lifespan extension (Figure 1).

An important finding of this study was miR-243’ role in enhancing proteostasis through activation of two major degradation pathways, known as Autophagy-Lysosome Pathway (ALP) and Ubiquitin-Proteasome System (UPS). Proteostasis is a cellular maintenance process in the organisms to degrade the misfolded and damaged proteins which generally accumulate with age and contribute to cellular dysfunction.^17^ The study revealed that upregulated miR-243 promoted the autophagic degradation of ubiquitin-tagged proteins by enhancing the expression of the autophagy receptor *sqst-3* and the lysosome-associated membrane protein *lmp-1*. The increased abundance of *sqst-3* and *lmp-1* proteins facilitates the formation of autolysosomes, important for the efficient degradation and removal of cellular debris. Additionally, miR-243 upregulated components of the SCF (Skp, Cullin, F-box protein) ubiquitin ligase complex, a multiprotein complex that enhances protein tagging for proteasomal degradation. This dual activation of ALP and UPS demonstrated miR-243‘s inclusive role in maintenance of protein quality control and mitigating proteotoxic stress, which is one of the hallmarks of healthy aging.

Furthermore, Kumar et al. suggested that miR-243 plays an important role in combating oxidative stress, a major regulator of cellular aging.^10^ miR-243 enhanced the expression of antioxidant gene *sod-3* which encodes the enzyme superoxide dismutase (SOD) that neutralizes reactive oxygen species (ROS), and thus, preserves the cellular integrity. Notably, *sod-3* is a transcriptional target of DAF-16 (a central regulator of miR-243), which further links miR-243 to the ILS signaling. The study also demonstrated that miR-243-mediated ROS mitigation not only protected the *C. elegans* against oxidative stress but also enhanced mitochondrial functions, as evidenced by reduced mitochondrial ROS production, elevated mitochondrial transmembrane potential, and increased ATP levels.

Kumar et al. also investigated the potential of postbiotic HK MKAK9 in improving immunity, thereby promoting worm’s defense against environmental and pathogenic stressors. The study showed involvement of p38 MAPK pathway in upregulating immune-related genes, such as *thn-1* and *ilys-1*, which enhance the worm’s ability to prevent infections. However, the direct involvement of miR-243 in immune resilience and its role in mitigating immune-induced cellular damage were not investigated in detail.

The study showed that interplay between miR-243 and ILS pathway (DAF-16-dependent) is specifically crucial in promoting longevity. By downregulating ILS pathway, miR-243 indirectly activates DAF-16 transcription factor, promoting a favorable environment conducive for managing the aging- and environment-induced stresses, as well as metabolic regulation (Figure 1). This modulation of the ILS pathway complements with the established longevity mechanisms observed in diverse species, where downregulated ILS/IGF-1 signaling has been linked with extended lifespan.

Kumar et al. highlights the therapeutic potential of targeting miR-243 for managing aging-associated neurodegenerative diseases like Alzheimer’s and Parkinson’s, as these are mainly characterized by protein aggregation. Thus, novel strategies to enhance miR-243 expression may improve proteostasis mechanisms and promote healthy aging in organisms. Microbiota-based interventions, including probiotic and postbiotic, may serve as noninvasive approach to upregulate miR-243 expression vis-à-vis to alleviate oxidative stress, enhanced immunity, and improve healthy state in aging populations. In summary, miR-243 emerges as a central regulator of stress resilience and aging in *C. elegans* through integrating proteostasis, stress resistance, and immune modulation into a cohesive framework. Linking microbe-derived signals to host cellular maintenance mechanisms and processes, Kumar et al. have laid the foundation for future research into miRNA-based therapeutics aimed at improving longevity and alleviating age-associated decline.

## Insights from Gabaldon et al.^10^: miR-243 and pathogen-induced diapause

Gabaldon et al. (2020) provided another interesting insight into the role of miR-243 in inducing pathogen-induced dauer formation (PIDF) in *C. elegans*.^11^ This study revealed that once worms are exposed to pathogens like *Salmonella enterica* serovar Typhimurium MST1 and *Pseudomonas aeruginosa* PAO1, these entered into a stress-resistant dauer stage, known as a protective mechanism against infections and environmental stressors (Figure 1).

Among the miRNAs analyzed, only miR-243 was consistently upregulated after two successive generations of pathogenic exposure, that indicates its important role in inducing PIDF. Notably, miR-243 mutant worms failed to induce the PIDF, irrespective of showing normal dauer formation under starvation conditions. This specificity indicated miR-243’s distinct role in pathogen-driven developmental processes and its importance in regulating stress-response pathways (Figure 1).

Gabaldon et al. showed that the regulation of miR-243 induced PIDF involves three key transcription factors such as DAF-16, PQM-1, and CRH-2, which are important in immune response and stress pathways in *C. elegans*. DAF-16 is known for its role in stress-induced nuclear translocation under stress condition and it activates crucial genes required for survival, such as dauer formation. PQM-1 influences DAF-16 activity, while CRH-2 is linked to immune modulation, therefore these transcription factors synergistically improve miR-243 expression during pathogen infection. This complex regulatory network leads to miR-243-facilitated PIDF as part of a fine-tuned immune-defense strategy in worms against pathogenic strains (Figure 1).

A notable finding of the study conducted by Gabaldon et al. are the transgenerational effects of miR-243. Exposure of pathogens in the parental generation leads to upregulation of miR-243 in the next generation as well with inherited intensified immune and stress resilience, a phenomenon attributed to transcriptional memory. This transgenerational adaptive memory supports long-term population resilience, highlighting the role of miR-243 as a molecular link between evolutionary survival strategies and environmental stressors. Notably, F1 generation exhibited a broad transcriptional change, while the F2 generation showed a more focused immune response, therefore indicating an adaptive refinement of defense mechanisms over generations (Figure 1). This phenomenon emphasizes miR-243’s role as a molecular regulator for environmental-based “learning” that allow *C. elegans* to optimize its defense strategy based on parental experiences.

Apart from the role of miR-243 in transcriptional regulation, Gabaldon et al. showed that miR-243 interacted with the RNA interference (RNAi) machinery through an argonaut protein RDE-1, suggesting that it induced gene silencing similar to siRNA pathways. This gene silencing mechanism most likely allows the downregulation of genes counterproductive to dauer formation and reinforces the protective response during PIDF.

Notably, the study identified high sequence similarity of miR-243 with genes such as *acs-2*, *C14B1.3*, and *mrp-2*, indicating potential targets for miR-243-mediated regulation. Future research should look into these miR-243-induced molecular cascades that regulate during pathogenic responses and its potential as a therapeutic target.

While activation of PIDF is advantageous for survival, but it comes with a reproductive cost, suggesting it to be a programmed survival strategy. Interestingly, the formation of dauer does not occur during the initial pathogenic encounters, rather it requires sustained exposure across generations. This is likely due to the progressive accumulation of environmental cues that allows upregulation of miR-243 and related transcriptional changes. This threshold-dependent regulation ensures entry to dauer state as a strategic decision, rather than involuntary event.

Gabaldon et al. also indicated the specificity of miR-243 compared to other small RNA’s, such as *let-7*, that is involved in broader immune responses. This distinction suggests the diverse roles of small RNAs in fine-tuning developmental and immune pathways in *C. elegans*.^11^ Overall, this study showed the role of miR-243 as a central regulator in adaptive responses to pathogenic infections and developmental response networks in *C. elegans* (Figure 1). Its regulation by crucial transcription factors, interaction with RNAi machinery, and transgenerational effects on stress resistance support its role in pathogen-induced survival strategy. This study not only provides insights into evolutionary mechanisms improving survival under adverse environments, but also indicates the potential of miR-243 as therapeutic target for stress-modulated conditions. However, further studies may shed light on molecular cascades regulated by miR-243 and its comprehensive involvement in stress resilience and other developmental processes across species.

## Unique features of miR-243 compared to other aging-related miRNAs

miR-243 has a unique characteristic of microbial adaptability, integrating environmental sensing with transgenerational immunity, that distinguishes it from other aging-related miRNAs in *C. elegans*.^10,11^ Unlike canonical aging-associated microRNAs such as *let-7*, miR-71, and miR-34, which primarily govern intrinsic aging, developmental timing, or stress responses, miR-243 specifically responds to microbial exposure and mediates heritable physiological outcomes.^2,3,10,11,18^ The well-conserved *let-7* family primarily regulates metabolic aging and larval-to-adult developmental timing, with modest roles in immunity, but its activity generally remains developmentally programmed and microbe-independent.^3^ While *let-7* is involved in broader immune responses, it lacks the microbial specificity of miR-243 in pathogen-induced developmental decisions.^3,11^ Similarly, miR-71/miR-228 influence longevity through neuroendocrine signaling, mainly affecting neuronal communication networks, but they do not distinguish between microbial species.^18,19^ In contrast, miR-243 functions as a downstream effector of microbial cues.^10,11^ miR-34 is recognized as a tumor suppressor and aging-related miRNA in mammalian systems, with roles in cellular senescence, DNA damage response, and p53 signaling.^3^ Although miR-34 is implicated in *Lacticaseibacillus rhamnosus* GG-mediated longevity and antibacterial defense, it lacks the microbial specificity and epigenetic heritability exhibited by miR-243.^3,10,11,20^ Unlike miR-34, which shows a generic but limited stress response, miR-243 responds broadly to both pathogens (*Salmonella*, *Pseudomonas*) and beneficial microbes (*Levilactobacillus brevis* MKAK9).^10,11^

The unique advantage of miR-243 is established by three major features. (1) **Dual proteostasis regulation**: unlike miR-34’s senescence-focused action, miR-243 simultaneously modulates autophagy–lysosome and ubiquitin–proteasome pathways for comprehensive protein quality control.^10^ (2) **Precise microbial specificity**: it discriminates between probiotics and pathogens, enabling context-dependent outcomes such as CRH-2- and PQM-1-regulated diapause or DAF-16-mediated longevity.^10,11^ (3) **Transgenerational epigenetic memory**: it uniquely mediates heritable stress resilience via HRDE-1, a feature absent in miR-34-regulated pathways.^11^ miR-243’s multi-tiered regulatory capacity connects microbial sensing, organismal stress adaptation, longevity, and intergenerational resilience. It thus provides horizontally dynamic regulation beyond the definitive, intrinsic roles of other aging-related miRNAs.

## Therapeutic implications and future directions

A key intersection of the studies by Kumar et al. and Gabaldon et al. indicates the role of miR-243 as a crucial regulator of DAF-16 transcription factor, which operates both proteostasis and stress adaptation. These studies provide complementary mechanistic insights into the biological functions of miR-243, that emerges as a crucial component of the organismal biological toolkit for stress adaptation and aging.^10,11^

Another interesting observation that emerges by comparing both the studies is that probiotic (heat-killed *L. brevis* MKAK9) and pathogens (*S. Typhimurium* MST1 and *P. aeruginosa* PAO1) both upregulated expression of miR-243, however led to distinct outcomes, i.e. increased lifespan versus dauer formation.^10,11^ This paradox might be explained by dose-dependent and tissue-specific effects of miR-243 expression in *C. elegans*. The duration, intensity, and tissue localization of induced miR-243 may vary greatly between probiotic and pathogenic exposures, where transient and moderate increase may facilitate protein homeostasis and longevity, while sustained, strong and systemic expression might lead to activation of survival response mechanisms like dauer formation. Moreover, miR-243 expression in intestine might primarily affect proteostasis and metabolic processes, while its neuronal expression could influence developmental decisions like dauer formation. This hypothesis may be supported by tissue-specific expression of DAF-16, which generally operates differentially in intestinal cells and neurons.^21^

**Figure 2.**
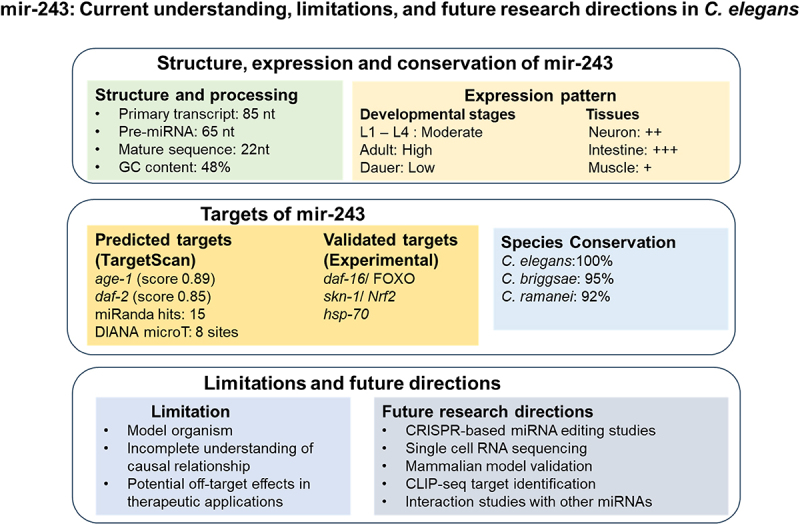
Regulatory roles of miR-243 in maintaining aging, cellular maintenance, and stress responses in *C. elegans*. This figure integrates the data from multiple sources, including WormBase,^22^ miRbase,^23^ and TargetScan^24^ to provide comprehensive knowledge about miR-243’s functions and significance. miR-243 is located on chromosome III (positions 13,245 –13,266) and functional or mature form generates 22-nucleotide long sequence with seed sequence (UGCGUUCAU). It is conserved microRNA with a remarkable evolutionary stability of 95% and 92% in *C. briggsae* and *C. remanei*, indicating its fundamental role in nematode physiology. The validated targets include DAF-16/FOXO transcription factors (with an 8mer binding site), molecular chaperone HSP-70 (6mer binding site), and the oxidative stress regulator SKN-1/Nrf2 (7mer-m8 binding site). Expression of miR-243 was high in adult stage and showed tissue-specific functions, mainly in neurons and intestinal cells. The predicted interactions showed high confidence score ( >0.8) for its regulatory role in the insulin/IGF-1 signaling and proteostasis pathways. Additionally, the figure shows limitation and future direction toward understanding the detailed mechanisms of miR-243.

The potential therapeutic interventions aimed at enhancing miR-243 expression could alleviate age-related diseases by improving efficient protein degradation, strengthen immune responses, and decreasing oxidative damage. Such therapeutics may also enhance pathogenic resistance and environmental stress tolerance, especially in aging populations. For instance, miR-243-targeted therapies may help in managing neurodegenerative diseases, such as Alzheimer’s and Parkinson’s by improving proteostasis and reducing protein aggregation. Likewise, miR-243’s role in immune modulation and oxidative stress resistance may support therapies for cardiovascular and metabolic disorders. miR-243 therapeutic potential lies not only in extending lifespan but also in enhancing quality of life during aging. Thus, future research should investigate miR-243’s conservation in higher organisms, particularly vertebrate models, to assess its translational potential (Figure 2).

While both the studies in this commentary focussed on linking miR-243 with the insulin-signaling pathway, future studies may investigate its cross-talk with other conserved pathways. For example, mTOR (mechanistic target of rapamycin), a central signaling pathway involved in cellular metabolism and growth, may show interaction with miR-243 by sharing their common downstream effector DAF-16.^25^ mTOR is considered as an important regulator of autophagy, which is a key cellular process for ensuring proteostasis by clearing damaged proteins and organelles,^25^ thereby improving cellular health and lifespan. Given that miR-243 promotes proteostasis by activating autophagy-lysosome pathway, it is possible that miRNA-243 may interact with mTOR pathway, either indirectly through its effect on DAF-16 activity or directly through targeting components of mTOR signaling pathway. Likewise, miRNA-243 may also interact with the AMP-activated protein kinase (AMPK) pathway, an evolutionary conserved energy sensor that generally extends longevity by mimicking the effects of calorie restriction and enhancing cellular stress resistance.^26^ AMPK pathway might be indirectly influenced by miR-243 through its effect on cellular energy homeostasis and mitochondrial function.^26^ Since Kumar et al. (2024) revealed the role of miR-243 in reducing oxidative stress and enhancing mitochondrial function, it is plausible that miR-243 may interact with the AMPK pathway, either through modulation of downstream effectors of AMPK signaling or by directly influencing the activity of AMPK. For example, miR-243 might influence the AMPK-dependent phosphorylation of DAF-16 transcription factor, which improves DAF-16 transcriptional activity and nuclear localization,^27^ thereby improving longevity and stress resilience.

High-throughput methods like CLIP-seq may help identify its molecular targets within key pathways such as ILS and p38 MAPK, and reveal its regulatory networks.^28^ In addition, advanced techniques like single-cell RNA sequencing and CRISPR-based miRNA editing could highlight its impact on specific developmental stages and tissues, thereby improving its therapeutic applications^29,30^ (Figure 2).

The interplay between miR-243 and environmental factors, including diet and microbiota may also warrant deeper investigations. Dietary interventions, including probiotic or postbiotics to increase miR-243 expression may serve as noninvasive strategy to promote longevity and healthy aging. Furthermore, understanding the interactions between other miRNAs with miR-243 could reveal antagonistic or synergistic mechanisms, explaining a broader regulatory network operating in longevity and stress resilience.

The intergenerational effect of miR-243 provides another intriguing therapeutic opportunity. It may be possible to enhance stress resilience to environmental stressors in offsprings by targeting miR-243 during maternal generation and potentially creating long-term healthy state across generations. This approach holds promise for ameliorating maternal health and evolutionary fitness through targeting miRNA modulation.

The study by Gabaldon et al. on transgenerational effects of miR-243 points out another question about the potential molecular carriers involved in transmitting this adaptive response. While their research did not directly examine these molecular carriers, but several hypotheses may be proposed. Firstly, the germline transmission of miRNAs may interact with PIWI-interacting RNA (piRNA) pathway (i.e. to maintain germline integrity), and result in transgenerational inheritance of adaptive traits.^31^ It is plausible that miR-243 might interact with the piRNA pathway, potentially by being incorporated into piRNA-containing ribonucleoprotein complexes for transmission to progeny or by affecting biogenesis or functionality of piRNAs. Secondly, HRDE-1, an argonaute protein may be involved in mediating transgenerational gene silencing in relation to environmental stresses.^31^ Given the role of miR-243 in the RNAi machinery through its interaction with RDE-1, as reported by Gabaldon et al. (2020), it is possible that miRNA-243 may interact with HRDE-1 or affect the synthesis of siRNAs which are bound by HRDE-1 required for transgenerational silencing of genes in stress responses. Thirdly, exosomes and other extracellular vesicles may be potential carrier of miR-243 and allow its shipping between different tissues and potentially intergenerational.^8^ Although limited studies in worms directly linked exosomes in transgenerational inheritance, but miR-243 may be packaged into these exosomes or extracellular vesicles, and transferred to progeny, potentially transmitting transgenerational immunity and stress resistance. Further, examining these extracellular vesicles for the presence of miR-243 would provide insight into detailed mechanism. Lastly, chromatin remodelling and histone modifications have also played role in intergenerational transfer of environmental experience-based adaptive responses in *C. elegans*.^32^ Epigenetic modifications, such as histone acetylation, methylation and chromatin remodelling, are key mechanisms for transgenerational inheritance in worms. It is possible that miR-243 may interact with these epigenetic factors by directly targeting genes encoding chromatin modifiers, or indirectly influencing signalling mechanisms regulating epigenetic processes. In addition, further research should also focus on investigating how these epigenetic mechanisms mediated miR-243 modulate transgenerational immunity, that may provide important insights into molecular basis of non-Mendelian inheritance. Together, miR-243 is emerging as a key central regulator of aging, immunity, and stress resistance, with potential therapeutic innovation. Its ability to orchestrate proteostasis, oxidative stress management, and intergenerational adaptation can be used as a cornerstone for developing therapeutic interventions to treat age-related diseases and promote healthy aging. Future studies aiming at uncovering its conserved roles in mammals may refine its clinical applications and undoubtedly provide the path for miRNA-based therapies that reevaluate aging and stress resilience in human health.

## Limitations and considerations

Despite the promising role of miR-243 in regulating lifespan and stress resilience of *C. elegans*, the studies by Kumar et al. and Gabaldon et al. have certain limitations that require careful considerations for translational relevance.^10,11^ The primary limitation is the current lack of direct experimental validation of miR-243-mediated aging and stress responses in mammalian systems. While *C. elegans* serves as an excellent model for fundamental aging mechanisms, the biological complexity of mammalian systems, including tissue-specific gene expression, organ-level interactions, and species-specific regulatory networks, may influence the functional outcomes of miR-243 modulation. However, several lines of evidence support the potential conservation and translational relevance of miR-243-related mechanisms. The miR-243 regulatory network demonstrates strong evolutionary conservation, particularly through the DAF-16/FOXO transcription factor pathway, which is highly conserved across species.^10,11^ Mammalian FOXO proteins (FOXO1, FOXO3, and FOXO4) play functionally similar roles in longevity, stress resistance, and proteostasis, suggesting conserved regulatory functions.^10^ Similarly, the insulin/IGF-1 signaling pathway targeted by miR-243 shows functional conservation from nematodes to humans, with well-established roles in aging and metabolic regulation.^10,11^ Furthermore, the cellular processes regulated by miR-243, including autophagy, proteasomal degradation, and oxidative stress response, represent fundamental mechanisms conserved across eukaryotes.^10,11^ The autophagy-lysosome pathway enhanced by miR-243 in *C. elegans* shares the core molecular machinery with mammalian autophagy, including conserved ATG proteins and lysosomal components.^10^ These evidences suggest that while direct mammalian validation remains necessary to confirm therapeutic potential, the strong evolutionary conservation of miR-243 target pathways provides a reasonable foundation for future translational research in higher organisms.

A critical knowledge gap regarding the function of miR-243 is the lack of direct evidence for its role as a major microbial sensor. Current evidence indicates that miR-243 acts as a downstream effector in microbial sensing cascades rather than functioning as a direct sensor.^10,11^ The increase in miR-243 expression after exposure to microbes likely involves upstream pattern recognition receptors (PRRs), such as Toll-like receptors or other receptors that detect conserved microbial signatures. These PRRs may activate transcriptional programs regulated by transcription factors like DAF-16, PQM-1, and CRH-2, which induce miR-243 expression.^10,11^ Future studies should aim to identify the precise upstream receptors and signaling cascades connecting microbial sensing to transcriptional activation of miR-243 gene, using chromatin immunoprecipitation and promoter analysis of the miR-243 gene locus. Additionally, the temporal expression profiling and pathway inhibition studies would help clarify the regulatory hierarchy controlling miR-243 expression.

A key limitation of these studies is their current inability to establish definitive cause and effect relationships. While both the studies observed a strong correlation between miR-243 upregulation and improved longevity and stress resilience, the complete spectrum of its molecular interactions and miR-243’s interaction remains to be elucidated. To confirm its functionality, the detailed, comprehensive studies focussed on direct manipulation of miR-243 levels under controlled conditions are essential. Such experimental investigations may help to explain its particular involvement in aging-related pathways and stress responses

Another critical consideration is the potential off-target effects in therapeutic applications. miRNAs, including miR-243, often regulate multiple genes, therefore raising the possibility of unexpected consequences when modulating their expressions. These potential off-target interactions could disrupt other critical cellular processes, which underline the need for rigorous evaluation of specificity and safety before using miR-243 as a therapeutic target for humans (Figure 2). Addressing these limitations and considerations through modern experimental techniques, such as high-throughput screening of miR-243 targets and CRISPR-based miRNA editing will be instrumental in refining its exact role (Figure 2). Additionally, the studies in mammalian models will further enhance the translational ability of miR-243 and development of effective and safe therapeutic approaches aimed at promoting healthy aging and stress resilience.

## Conclusion

miR-243 has emerged as an important regulator of longevity and stress resilience in *C. elegans* that is mediated by activation of pathways involved in enhancement of proteostasis, immune responses, and alleviation of environmental stress. Kumar et al. (2024) suggested its roles in regulating insulin-like signalling (DAF-16-dependent manner) and proteostasis, thereby promoting healthy aging and cellular stability under stress. Complementing this study, Gabaldon et al. (2020) showed miR-243’s ability to enhance immunity in the offspring, environmental adaptability, and transgenerational effects, underscoring its evolutionary significance. Together, these studies indicate miR-243’s potential as a versatile central regulator that balances internal cellular maintenances and external stress adaptation to optimize longevity and survival. Therapeutically, miR-243 promise significant potential for addressing age-related diseases like neurodegenerative disorders, by reducing protein aggregation and preventing oxidative stress in cardiovascular and metabolic conditions, with pending validation of its conservation and function in mammalian systems. Dietary and probiotic strategies targeting miR-243 may offer noninvasive approaches to improve healthspan. Future studies should focus on investigating its precise molecular targets, tissue-specific effects, and conservation across species, particularly in mammalian models, before advancing toward clinical applications. miR-243 provides a promising avenue for its innovative role in aging therapies and transgenerational health benefits.

## Data Availability

No new data were generated during the preparation of this publication; therefore, no data sharing statement is applicable.
